# Data Resource Profile: Victorian Comprehensive Cancer Centre Data Connect

**DOI:** 10.1093/ije/dyad148

**Published:** 2023-10-27

**Authors:** Alex Lee, Damien McCarthy, Rebecca J Bergin, Allison Drosdowsky, Javiera Martinez Gutierrez, Chris Kearney, Sally Philip, Meena Rafiq, Brent Venning, Olivia Wawryk, Jianrong Zhang, Jon Emery

**Affiliations:** Department of General Practice, Faculty of Medicine, University of Melbourne and Centre for Cancer Research, Parkville, VIC, Australia; Department of General Practice, Faculty of Medicine, University of Melbourne and Centre for Cancer Research, Parkville, VIC, Australia; Department of General Practice, Faculty of Medicine, University of Melbourne and Centre for Cancer Research, Parkville, VIC, Australia; Department of General Practice, Faculty of Medicine, University of Melbourne and Centre for Cancer Research, Parkville, VIC, Australia; Department of General Practice, Faculty of Medicine, University of Melbourne and Centre for Cancer Research, Parkville, VIC, Australia; Department of Family Medicine, School of Medicine, Pontificia Universidad Católica de Chile, Santiago, Chile; Department of General Practice, Faculty of Medicine, University of Melbourne and Centre for Cancer Research, Parkville, VIC, Australia; Department of General Practice, Faculty of Medicine, University of Melbourne and Centre for Cancer Research, Parkville, VIC, Australia; Department of General Practice, Faculty of Medicine, University of Melbourne and Centre for Cancer Research, Parkville, VIC, Australia; Epidemiology of Cancer and Healthcare Outcomes (ECHO) Group, UCL, London, UK; Department of General Practice, Faculty of Medicine, University of Melbourne and Centre for Cancer Research, Parkville, VIC, Australia; Department of General Practice, Faculty of Medicine, University of Melbourne and Centre for Cancer Research, Parkville, VIC, Australia; Department of General Practice, Faculty of Medicine, University of Melbourne and Centre for Cancer Research, Parkville, VIC, Australia; Department of General Practice, Faculty of Medicine, University of Melbourne and Centre for Cancer Research, Parkville, VIC, Australia

**Keywords:** Primary care data, data linkage, cancer services research

Key Features

- Data Connect has been established as a data linkage resource that brings together Australian General Practice (GP), Metropolitan Hospital and Cancer Registry data sources to enable researchers to address questions in health services research related to the entire patient continuum of care.
- This resource includes patient-level de-identified data on over 3 million Victorian primary care patients aged over 18 years of age, with GP data covering the period 1996–2021 and hospital data covering the years 2006–21.
- Data have been linked across health services as this is a requirement for understanding patients’ journey of care, in particular for cancer patients, who interact with many different parts of the health care system, often starting at primary care.
- The resource includes rich clinical and administrative data from hospital presentations and admissions, GP encounters, prescriptions, pathology test results and observations and cancer diagnoses.
- Researchers interested in using this resource should contact Data Connect at [https://vcccalliance.org.au/our-work/research-and-translation/data-connect].

## Data resource basics

### Victorian Comprehensive Cancer Centre Data Connect

Victorian Comprehensive Cancer Centre (VCCC) Data Connect is a single platform for facilitating access to a range of Victorian health data sources, allowing researchers, analysts and policy makers to bring a rigorous, data-driven lens to questions related to health services and patterns of cancer care. It has been developed in Australia since 2017 to connect general practice databases with data from hospitals and clinical cancer registries, and involves nearly 3 million patients spanning the years 1996–2021. It is updated on a yearly basis to incorporate more recent datasets, and is being expanded to include additional health services datasets across the state, including from non-metropolitan hospitals, VCCC Alliance members and clinical registries. In this paper we provide high-level descriptive statistics of the data sources, summarizing representativeness, data quality and overall sizes of patient cohorts. As a specific example we then focus on general cancer statistics, to illustrate the utility of this resource.

Cancer patients interact with the health system through using many different health services, with primary care playing a critical role from initial presentation through to survivorship and end-of-life care.^1^^,^^2^ Each health service has its own corresponding data collection systems, so linkage is critical to understand how patients interact with the system across their entire journey of care.^3^

In Australia, health data linkage is challenging due to different parts of the health system being the responsibility of different jurisdictions at state and federal levels, as well as due to differing data standards and systems.^4^ Since primary care is usually the first point of contact that patients have with the health system,^5^ including prior to a diagnosis of cancer, General Practice (GP) data are a key component of research in this area.

The intended audience of this paper is researchers interested in data, research and analysis related to health service use. Research studies using these data are already under way, including in the study of times to diagnosis and treatment, pathways of care, early detection of cancer and cancer survivorship. Whereas our particular focus is on cancer, Data Connect contains a wealth of information on other cohorts of patients with broader applications, and the resource is of value for health services researchers in different clinical domains.

### Other linked data resources

Linked data resources are becoming increasingly important for health research in many countries.^6–8^ In the UK, the Clinical Practice Research Datalink (CPRD) is one such resource, covering almost 7% of the UK population, and has been used in over 1000 studies.^6^ In Australia, state and federal governments have their own linked data infrastructure. Examples include the Centre for Victorian Data Linkage,^9^ Data Linkage Western Australia,^10^ the Australian Institute of Health and Welfare^11^ and also the Australian Bureau of Statistics’ own linkage capabilities.^12^ However in Australia, primary care is often overlooked in linked data research, despite its critical role in the health system.^4^^,^^5^ For example, in the 2020–21 financial year, over 85% of Australians had at least one contact with a GP service.^13^ A key source of general practice data is the MedicineInsight database, a representative database covering the entire country.^14^ Another is the University of Melbourne Patron Data for Decisions Program.^4^ Few studies have involved linking health services datasets to these types of general practice data sources that contain rich clinical information in addition to billing information.^15^

### Data governance, ethics, privacy and security

Data access is provided by BioGrid Australia,^16–18^ a not-for-profit organization that provides an online platform for data linkage and management, so data storage and linkage infrastructure are handled by a single entity. Data linkage in Data Connect is carried out through the use of a Unique Subject Identifier (USI), a unique encrypted key for each patient. For hospital data these USI values are generated by processing patient-identifying information through BioGrid’s probabilistic record linkage key software and using the GRHANITE^TM^ data linkage software,^19^ whereas for GP data these are generated at the practice using GRHANITE^TM^. The USI effectively anonymizes patient records as no identifying information is released to researchers, protecting patient privacy and ensuring that data governance, ethics and privacy requirements of data custodians that are met.

Data Connect uses the BioGrid federated data infrastructure, which means that it is covered by BioGrid’s data governance model.^18^ A collaboration agreement among BioGrid member institutions ensures that there is no need to create multiple agreements for different health service sites in order to apply for data access.^16^ In addition, the human research ethics framework is managed by BioGrid, so that data access, governance and management across sites are covered by a single agreement, removing the need for researchers to approach data custodians individually and ensuring more timely access to data for research. It is important to note that data custodian and ethical approval is required for each research project prior to linked data being made available to researchers through BioGrid.

**Figure 1.**
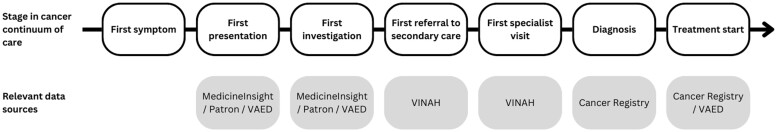
Stages in the continuum of care and relevant data sources. Patron, University of Melbourne general practice database; VAED, Victorian Admitted Episodes Dataset; VINAH, Victorian Integrated Non-Admitted Health dataset

Data linkage allows researchers, analysts and policy makers to capture a more complete picture of patients’ interactions with the health care system.^3^ Primary care has a critical role through the patient’s continuum of care,^1^^,^^2^ as illustrated in the Figure 1, and linking data from general practice, hospitals and clinical registries allows tracking of patients along this continuum.

**Figure 2.**
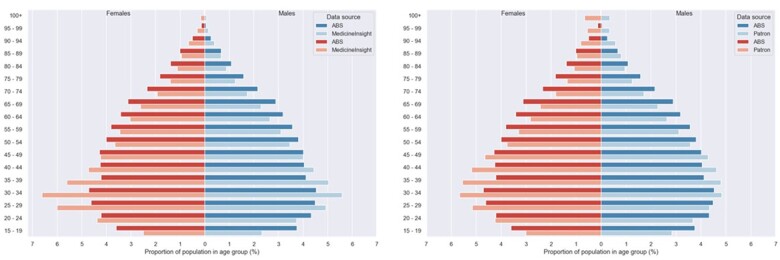
Distribution of patients by age and sex in MedicineInsight vs 2016 Census (left) and Patron vs 2016 Census (right). ABS, Australian Bureau of Statistics; Patron, University of Melbourne general practice database

Figure 1 illustrates the relation between points along the cancer continuum of care and corresponding datasets that are available through Data Connect. In this case we provide the example of a patient presenting to general practice with a symptom, and points along their care pathway until diagnosis and treatment. Different events are captured in different electronic health records systems, so linkage is crucial to fully understand patients’ interactions.

Questions related to early diagnosis of cancer involve general practice and hospital data, and questions related to survivorship relate to both earlier and later stages.

## Data collected

Data Connect currently contains the following datasets:

- MedicineInsight: General Practice database (Victorian subset): MedicineWise^14^^,^^20^;
- VAED: Victorian Admitted Episodes Dataset (four hospital networks);
- VEMD: Victorian Emergency Minimum Dataset (three hospital networks);
- VINAH: Victorian Non-Admitted Episodes Health dataset (two hospital networks);
- Patron: University of Melbourne general practice database^4^;
- VCR Pilot: A sample of patients from the Victorian Cancer Registry;
- ACCORD: Australian Comprehensive Cancer Outcomes and Research Database (colorectal and sarcoma subsets);
- AURORA: AUstralian Registry and biObank of thoRAcic cancers.

Additional work is under way to link radiotherapy data, additional cancer registry data and genomics datasets. The datasets contain patient demographic information as well as data related to patient interactions with health services. Table 1 provides a more detailed (though not exhaustive) list of information available. We also highlight certain items that are not available, mainly due to privacy. Further detailed information on each of these is available in the references. Note that the National Prescribing Service ceased operation at the end of 2022 and the primary care database is now managed by the Australian Commission on Safety and Quality in Health Care.

**Table 1.** Summary of data sources

Dataset	Source of data	Variables available (not exhaustive)	Variables not available (not exhaustive)
**MedicineInsight** (General Practice database, Victorian subset)	MedicineWise	Encounter date Patient residence (at SA3) Patient age Patient sex Health care card status Alcohol status Smoking status ATSI status Requested tests Test results Prescriptions Billing Observations such as weight and height measurements Reason for encounter, including signs and symptoms Conditions Diagnoses^a^ Comorbidities are captured in the reason for encounter, conditions and diagnoses free-text fields	Clinical notes Time of encounter Practice location Patient ethnicity Patient country of birth
**Patron** (University of Melbourne general practice database)	Practices recruited via University of Melbourne Data for Decisions Program	Encounter date Patient residence (at SA3) Patient age Patient sex Health care card status Alcohol status Smoking status ATSI status Requested tests Test results Prescriptions Billing Observations such as weight and height measurements Reason for encounter, including signs and symptoms Conditions Diagnoses^a^ Comorbidities are captured in the reason for encounter, conditions and diagnoses free-text fields	Clinical notes Time of encounter Practice location Patient ethnicity Patient country of birth
**VAED** (Victorian Admitted Episodes dataset)	Victorian public hospitals via BioGrid	Date of admission Admission site ICD-10 diagnosis Diagnosis date Separation dates Patient age Patient sex CALD indicators	Clinical notes
**VEMD** (Victorian Emergency Minimum Dataset)	Victorian public hospitals via BioGrid	Presentation date Presentation site Patient postcode CALD indicators	Clinical notes
**VINAH** (Victorian Integrated Non-Admitted Health dataset)	Victorian public hospitals via BioGrid	Episode date Episode site Patient postcode	Clinical notes
**Victorian Cancer Registry Pilot** (Sample of patients from the Victorian Cancer Registry)	Cancer Council Victoria	Diagnosis date Patient age Patient sex Patient date of birth Patient country of birth Patient residence (at SA2) Cancer diagnosis code (ICD-10-AM) Tumour site Tumour stream	SA1 geography Limited stage data
**AURORA** (AUstralian Registry and biObank of thoRAcic cancers)	Peter MacCallum Cancer Centre	Diagnosis date Treatment date Patient age Patient sex Tumour stage Treatment type and usage Comorbidities Date of death	Patient residence Limited symptom data
**ACCORD Colorectal** (Australian Comprehensive Cancer Outcomes and Research Database, colorectal subset)	Royal Melbourne Hospital and Western Hospital	Diagnosis and treatment dates Patient age Patient sex Patient weight Tumour stage Chemotherapy drugs used Dose Radiotherapy fractions Reasons for changes in dose Resections Comorbidities Cancer history	Patient residence Limited symptom data
**ACCORD Sarcoma** (Australian Comprehensive Cancer Outcomes and Research Database, sarcoma subset)	Peter MacCallum Cancer Centre	Patient age Patient sex Cancer history Death date Referring specialty First consultation details Symptom history Pre-treatment imaging status Diagnosis and treatment dates Tumour stage Biopsy details Resection details Surgery details Radiotherapy details Chemotherapy details	Limited symptom data

ATSI, Aboriginal and Torres Strait Islander; CALD, culturally and linguistically diverse; ICD-10, International Classification of Diseases, Tenth Revision; ICD-10-AM, International Classification of Diseases, Tenth Revision, Australian Modification; SA1, Statistical Area Level 1, from the Australian Bureau of Statistics Australian Statistical Geography Standard [https://www.abs.gov.au/statistics/standards/australian-statistical-geography-standard-asgs-edition-3/jul2021-jun2026/main-structure-and-greater-capital-city-statistical-areas/statistical-area-level-1]; SA2, Statistical Area Level 2, from the Australian Bureau of Statistics Australian Statistical Geography Standard [https://www.abs.gov.au/statistics/standards/australian-statistical-geography-standard-asgs-edition-3/jul2021-jun2026/main-structure-and-greater-capital-city-statistical-areas/statistical-area-level-2]; SA3, Statistical Area Level 3, from the Australian Bureau of Statistics Australian Statistical Geography Standard [https://www.abs.gov.au/statistics/standards/australian-statistical-geography-standard-asgs-edition-3/jul2021-jun2026/main-structure-and-greater-capital-city-statistical-areas/statistical-area-level-3].

a These are recorded in free-text and mapped by MedicineWise. They are not based on the International Classification of Primary Care (ICPC) standard.

### Data quality

Table 2 summarizes some general data quality issues in the datasets that make up Data Connect. More detailed data quality work for each of the data sources can be found in the references. In any case, for a given project, data quality assessment should be first carried out based on the specific research question as part of feasibility assessment. The statistics reported here should help researchers understand some of the major data quality issues that occur generally. Note that both MedicineInsight and Patron are drawn from similar Electronic Medical Record (EMR) systems—Best Practice^21^ and Medical Director^22^—with Patron also containing data from ZedMed.^23^ As a result, the data are similar, with similar issues and limitations. Around 580 000 patients are common to Patron and MedicineInsight.

**Table 2.** A non-exhaustive list of data quality issues

Dataset	Variable	Result	Specific data quality issues
MedicineInsight, Patron	Reason for encounter	A large proportion of these are free-text; drop-down boxes are from bespoke medical terminology lists, rather than standardized ones	Work needs to be done to map the ‘reason for encounter’ fields to standard medical terminology; this is under way for the Patron database
	Age	Differences in age distribution between GP data and census	
	Sex	More women than men represented	
	SEIFA	Distribution of patients agrees well with Census 2016^12^	This is only reported at SA3 level which leads to some issues with interpretation^20^
	Indigenous status	Not a reliable variable; does not agree with 2016 Census	Often not recorded
	SA3 of patient residence	For MedicineInsight counts agree well with those reported in Census 2016^12^	Corresponds to SA3 boundaries for 2020; patient may have changed residence
	Conditions	Data regarding time a condition was diagnosed is often unreliable	
VAED	Local Government Area catchment	Not representative	Currently concentrated in metropolitan Melbourne
VEMD	Local Government Area catchment	Not representative	Currently concentrated in metropolitan Melbourne
VINAH	Local Government Area catchment	Not representative	Currently concentrated in metropolitan Melbourne
VCR Pilot	SA2	Not representative	Contains only a random sample of data that can be linked to Royal Melbourne Hospital and no other hospitals
AURORA	Stage at diagnosis	Not representative if only using the data from one centre	Data from Peter MacCallum Cancer Centre are predominantly for late-stage disease; data from St Vincent’s Hospital are predominantly for early-stage disease
ACCORD Colorectal	Cancer history	High percentage of unknown and/or missing data	Previous cancer diagnosis information not available

ACCORD, Australian Comprehensive Cancer Outcomes and Research Database (colorectal subset); AURORA, AUstralian Registry and biObank of thoRAcic cancers; MedicineInsight, General Practice database (Victorian subset); Patron, University of Melbourne general practice database; SA3, Statistical Area Level 3; SA2, Statistical Area Level 2; SEIFA, Socio-Economic Index For Areas; VAED, Victorian Admitted Episodes dataset; VCR Pilot, a sample of patients from the Victorian Cancer Registry; VEMD, Victorian Emergency Minimum Dataset; VINAH, Victorian Integrated Non-Admitted Health dataset.

**Table 3.** Data linkage statistics of data that make up Data Connect

Dataset	Description	Coverage	Number of unique patients	Information
MedicineInsight	General practice dataset covering patients from 92 Victorian general practices	1/1/2007–31/12/2017	1.39 M	Encounters: 30 M Diagnoses: 9.6 M Prescriptions: 13.1 M Observations: 33.7 M Pathology results: 89.3 M Requested tests: 7 M Billing events: 20.6 M
Patron	General practice dataset covering patients from 130 Victorian general practices	∼4/1/1996–30/11/2020	1.56 M	Encounters: 42.7 M Diagnoses: 8.4 M Prescriptions: 40 M Observations: 40 M Pathology results: 105 M Requested tests: 8M Billing events: 32.8M
VAED	Patients admitted to hospital (four hospitals)	2006/07–2020/21 (financial year)	887 629	Admitted episodes: 4.2 M Diagnoses: 4.2 M Procedures: 7.8 M
VEMD	Patients presenting at a hospital emergency department (three hospitals)	2006/07–2019/20 (financial year)	947 229	Emergency presentations: 2.7 M
VINAH	Patients using outpatient services (2 hospitals)	2012/13–2019/20 (financial year)	380 424	Episodes: 1.1 M Contacts: 3.1 M
VCR (Sample)	Victorian Cancer Registry (Pilot): a sample of patients from Royal Melbourne Hospital	86% between 1/1/2012 and 27/12/2018; remainder go back to 1982	16 301	Cancer diagnoses: 20 978
ACCORD Colorectal	Hospital Cancer Registry data for colorectal cancer patients from Royal Melbourne Hospital, Western Health and Eastern Health	1988–2021	10 984	Colorectal cancer diagnoses: 10 984
ACCORD Sarcoma	Hospital Cancer Registry data for Sarcoma tumours from Peter MacCallum Cancer Centre	1980–2021	3746	Sarcoma diagnoses: 3746
AURORA	Hospital Cancer Registry data for thoracic tumours for patients from Peter MacCallum Cancer Centre, St Vincent’s Hospital and others in Victoria	1984–2021	2716 from Peter MacCallum Cancer Centre, including 867 linked to MedicineInsight or Patron	764 patients with at least one encounter pre-diagnosis (420 with at least one GP encounter in the 12 months prior to diagnosis)

ACCORD, Australian Comprehensive Cancer Outcomes and Research Database (colorectal subset); Australian Comprehensive Cancer Outcomes and Research Database (sarcoma subset); AURORA, AUstralian Registry and biObank of thoRAcic cancers; MedicineInsight, General Practice database (Victorian subset); Patron, University of Melbourne general practice database; VAED, Victorian Admitted Episodes dataset; VCR Sample, a sample of patients from the Victorian Cancer Registry; VEMD, Victorian Emergency Minimum Dataset; VINAH, Victorian Integrated Non-Admitted Health dataset.

### Coverage and representativeness

More detailed statistics of datasets in Data Connect are shown in Table 3. Geographical coverage, described in Table 4, is greater for the general practice datasets, covering all parts of Victoria, with less coverage in the eastern part of metropolitan Melbourne. In 2019 there were 1985 general practices in Victoria,^24^ so approximately 10% of general practices in Victoria are represented. For the hospital datasets, no hospitals outside the metropolitan area are currently available.

**Table 4.** Geographical coverage

Dataset	Geographical coverage	Demographic representativeness
MedicineInsight	All of Victoria, though under-represented in the east	Similar to Victorian population across sex, age and SEIFA
Patron	All of Victoria	Similar to Victorian population across sex, age and SEIFA
VAED	Higher representation in metropolitan Melbourne	Patients mainly from Metropolitan Melbourne
VEMD	Higher representation in metropolitan Melbourne	Patients mainly from Metropolitan Melbourne
VINAH	Higher representation in metropolitan Melbourne	Patients mainly from Metropolitan Melbourne
VCR	Higher representation in metropolitan Melbourne	Only those patients who can be linked to hospitals from the VAED

MedicineInsight, General Practice database (Victorian subset); Patron, University of Melbourne general practice database; SEIFA, Socio-Economic Index For Areas; VAED, Victorian Admitted Episodes dataset; VCR, a sample of patients from the Victorian Cancer Registry; VEMD, Victorian Emergency Minimum Dataset; VINAH, Victorian Integrated Non-Admitted Health dataset.

Figure 2 shows the age distribution of patient populations in Patron and MedicineInsight. MedicineInsight data closely matches the broader Victorian population in terms of distribution of age and sex,^14^ though there are a larger proportion of younger patients, particularly female patients. This is consistent with other studies showing that women are more likely to visit a GP.^25^ Ages below 18 are not shown as there is no current ethical approval to include under-18s in Data Connect. For example, BioGrid receives under 18s data from MedicineInsight, but we filter these patients out as there is no current ethical approval to include children.

### Types of cancers and prevalence within the linked data

Cancer diagnoses are recorded in registries as well as hospital admission episodes data. From this, the prevalence of different types of cancers in a specific patient cohort can be calculated: in this case, patients who were admitted to one of the four metropolitan hospitals for which data are available through Data Connect. A total of 79 277 patients are diagnosed with cancer in VAED data, 8.9% of the total.^26^ The ICD-10-AM (International Classification of Diseases, Tenth Revision, Australian Modification) codes have been used to filter to the specific cancer stream. The results are shown in Table 5.

**Table 5.** Cancer types and counts of patients

Body system	ICD-10-AM codes	Number of patients	% of patients
Genitourinary	C60-C68	12 152	15.3%
Clinical haematology	C81-C86, C88, C90-C96, D45-D47	11 794	14.9%
Melanoma and skin	C43-C44	11 128	14.0%
Lung	C33-C34, C38-C39, C45	9211	11.6%
Lower gastrointestinal	C17-C21, C26	8380	10.6%
Upper gastrointestinal	C15-C16, C22-C25	7881	9.9%
Breast	C50	7205	9.1%
Head and neck	C00-C14, C30-C32, C37, C69, C73	4122	5.2%
Secondary/other	C74-C80	2779	3.5%
Neuro-oncology	C70-C72	2089	2.6%
Gynae-oncology	C48, C51-C58	1930	2.4%
Sarcoma	C40-C41, C46-C47, C49	557	0.7%
Unknown primary	C80	49	0.1%

ICD-10-AM, International Classification of Diseases, Tenth Revision, Australian Modification.

### Data structure and extraction

The GP data is extracted from three Electronic Medical Record systems: Best Practice; Medical Director; and ZedMed. The data linkage software GRHANITE^TM^ is installed at each of the contributing practices. Hospital data sources are extracted from the EMR systems at Metropolitan Melbourne hospitals and are then provided to BioGrid for linkage.

## Data resource use

There are many studies currently under way that make use of Data Connect. The focus of each involves a different part of the continuum of care for cancer patients. Two examples include the following.

- Diagnostic intervals: time to diagnosis is an important factor driving survival rates of cancer patients.^27^^,^^28^ Using data linked between MedicineInsight, Patron and the AURORA and ACCORD clinical registries, researchers have been studying diagnostic intervals for lung (268 patients) and colorectal cancers (273 patients).
- Primary care blood test use and early detection of cancer: in the 6 months leading up to a diagnosis of cancer, population-level trends in pathology tests and signs and symptoms become apparent. These can facilitate the early detection of cancer through assisting GPs to focus on those clinical features that are most likely to be predictive of cancer.

Finally, there is currently a lack of standards in Australia for defining phenotypes from electronic health records. Phenotyping libraries are publicly accessible catalogues of disease and other health-related definitions, which ensure reproducibility of research outputs. In the UK, Health Data Research (HDR) UK^29^ has produced an example of such a library. PheKB (the Phenotype Knowledge Base) developed in the USA is another example.^30^ A recent review defined a set of desiderata for a high-quality phenotype library.^31^ Due to differences in the way that electronic health care data are recorded in Australia, a similar resource would be very valuable here as well. This would provide a way for researchers to share reusable definitions that appear in observational studies involving Victorian health records, and would better ensure reproducible, high-quality research outputs.

## Strengths and weaknesses

### Strengths

Key strengths of Data Connect include the detailed unit-record-level data on over 3 million patients in Victoria and its broad applicability to a range of questions in health services research. It brings together general practice, hospital and clinical registry data and is particularly valuable for studying the entire patient journey from a patient’s first presentation at a health service through to diagnosis, treatment, survivorship and end-of-life care. Rich clinical information is available about test results, observations, signs, symptoms, dates of events, locations and demographic information such as age, sex and place of residence, allowing study of many different questions involving all stages in the continuum of cancer care. The examples of types of questions we are examining in colorectal cancer demonstrates the utility of these linked datasets.

### Weaknesses

Some limitations of Data Connect include the following.

- The quality and quantity of data varies across different variable types and between datasets, which may limit the feasibility of certain studies particularly, for example, less common cancers.
- Currently only some hospitals in metropolitan Melbourne are represented and over 200 general practice sites provide data. This is just over 10% of those in Victoria.^24^ There are two arising limitations: incomplete data on hospitalizations and emergency presentations, and limited size of cohorts for which general practice data can be linked.
- More generally in relation to cohort size, although there are many patients represented across all datasets, for a given cohort this number is significantly smaller, as demonstrated in the colorectal cancer example. In comparison with jurisdictions such as the UK, Victoria’s population is much smaller, so this can be a limitation to studies requiring larger sample sizes, for example where survival may be the outcome of interest for investigating specific subgroups.

## Data resource access

**l**For further information about access to Data Connect data, please contact Sally Philip, Program Manager, VCCC Data Connect, and Alex Lee, Research Fellow, via the Data Connect website at [vcccalliance.org.au/our-work/research-and-translation/data-connect/contact-us].

## Ethics approval

This work was approved by Melbourne Health HREC.
